# Protocols for RecET‐based markerless gene knockout and integration to express heterologous biosynthetic gene clusters in *Pseudomonas putida*

**DOI:** 10.1111/1751-7915.13374

**Published:** 2019-02-14

**Authors:** Kyeong Rok Choi, Sang Yup Lee

**Affiliations:** ^1^ Metabolic and Biomolecular Engineering National Research Laboratory Systems Metabolic Engineering and Systems Healthcare Cross Generation Collaborative Laboratory Department of Chemical and Biomolecular Engineering (BK21 Plus Program) Institute for the BioCentury Korea Advanced Institute of Science and Technology (KAIST) 291 Daehak‐ro Yuseong‐gu Daejeon 34141 Republic of Korea; ^2^ BioProcess Engineering Research Center KAIST 291 Daehak‐ro Yuseong‐gu Daejeon 34141 Republic of Korea; ^3^ BioInformatics Research Center KAIST 291 Daehak‐ro Yuseong‐gu Daejeon 34141 Republic of Korea; ^4^ Novo Nordisk Foundation Center for Biosustainability Technical University of Denmark 2800 Kongens Lyngby Denmark

## Abstract

*Pseudomonas putida* has emerged as a promising host for the production of chemicals and materials thanks to its metabolic versatility and cellular robustness. In particular, *P. putida* KT2440 has been officially classified as a generally recognized as safe (GRAS) strain, which makes it suitable for the production of compounds that humans directly consume, including secondary metabolites of high importance. Although various tools and strategies have been developed to facilitate metabolic engineering of *P. putida*, modification of large genes/clusters essential for heterologous expression of natural products with large biosynthetic gene clusters (BGCs) has not been straightforward. Recently, we reported a RecET‐based markerless recombineering system for engineering *P. putida* and demonstrated deletion of multiple regions as large as 101.7 kb throughout the chromosome by single rounds of recombineering. In addition, development of a donor plasmid system allowed successful markerless integration of heterologous BGCs to *P. putida* chromosome using the recombineering system with examples of – but not limited to – integrating multiple heterologous BGCs as large as 7.4 kb to the chromosome of *P. putida* KT2440. In response to the increasing interest in our markerless recombineering system, here we provide detailed protocols for markerless gene knockout and integration for the genome engineering of *P. putida* and related species of high industrial importance.

## Graphical abstract

*Pseudomonas putida* is a promising engineering host for the production of chemicals including secondary metabolites, but engineering tools for integration of large biosynthetic clusters of such valuable compounds to *P. putida* chromosome have been time‐consuming or uncontrollable. Recently, we reported a RecET‐based markerless recombineering system for *P. putida* and demonstrated knocking out chromosomal regions as large as 101.7 kb and integration and subsequent successful expression of multiple heterologous biosynthetic genes and clusters in *P. putida*. Detailed protocols for the markerless recombineering system for *P. putida* is provided.

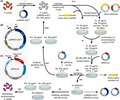

## Introduction

Cellular robustness and versatile metabolism of *Pseudomonas putida* have characterized this Gram‐negative soil bacterium as an attractive workhorse of metabolic engineering for the bio‐based production of chemicals and materials (Nikel *et al*., 2016; Nikel and de Lorenzo, 2018). Particularly, *P. putida* KT2440 has been officially classified as a generally recognized as safe (GRAS) strain (Federal Register, 1982) and exploited for the production of chemicals and products that human directly consumes, especially natural products including pharmaceuticals, nutraceuticals, and cosmetic ingredients (Loeschcke and Thies, 2015). Various genetic engineering elements – including counterselection markers (Galvao and de Lorenzo, 2005; Gross *et al*., 2006; Graf and Altenbuchner, 2011; Johnson *et al*., 2017), site‐specific recombinases (Leprince *et al*., 2012; Ibrahim *et al*., 2015), homing endonuclease I‐SceI (Martinez‐Garcia and de Lorenzo, 2011; Martinez‐Garcia *et al*., 2014; Chen *et al*., 2016), bacteriophage‐derived recombinases homologous to Bet protein from λ Red system (Aparicio *et al*., 2016; Luo *et al*., 2016), and clustered regularly interspaced palindromic repeat (CRISPR)/CRISPR‐associated (Cas) systems (Aparicio *et al*., 2017; Cook *et al*., 2018; Sun *et al*., 2018) – have been incorporated to facilitate gene knockout in *P. putida*. Although reduction of the genome size can be beneficial in some cases for better strain performance (Lieder *et al*., 2015), only few cases of deleting large genomic fragment have been reported (Martinez‐Garcia *et al*., 2014; Aparicio *et al*., 2017). In addition, integration of heterologous biosynthetic gene clusters (BGCs) to *P. putida* chromosome for the production of heterologous secondary metabolites has relied on time‐consuming homologous recombination with selection markers (Wenzel *et al*., 2005; Gross *et al*., 2006; Cao *et al*., 2012; Gong *et al*., 2016) and unpredictable transposon‐mediated random insertion (Glandorf *et al*., 2001; Chai *et al*., 2012; Loeschcke *et al*., 2013; Domrose *et al*., 2015, 2017), requiring the development of rapid and reliable integration system for programmed introduction of heterologous BGCs to *P. putida* chromosome.

**Figure 1:**
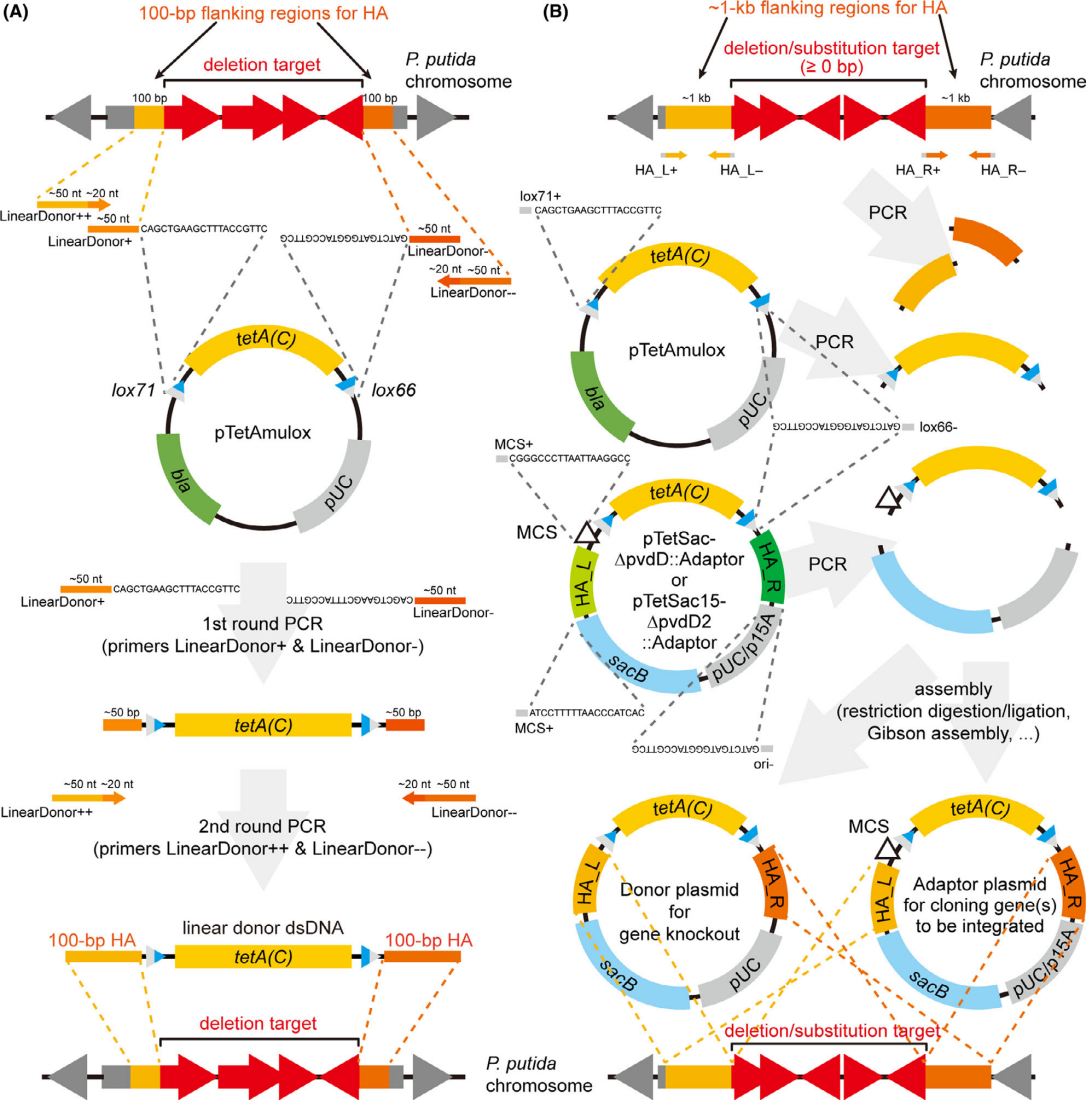
Design of homology arms (HAs) and construction of donor DNAs. A. Scheme of designing 100‐bp HAs and primers for the construction of linear donor dsDNAs. B. Scheme of designing ~1‐kb HAs and primers for the construction of donor plasmids for gene knockout and adaptor plasmids for cloning genes to be integrated. Dashed lines in (A) and (B) indicate sequence homologies and directionality among different constructs. Names of primers mentioned in the protocols (Table S3) are presented next to the primers with some fixed sequences, if available. *bla*, ampicillin resistance gene; *tetA(C)*, tetracycline resistance gene; HA_L & HA_R, HAs placed left and right to the *tetA(C)* gene; MCS, multiple cloning site. pUC, pUC origin of replication; p15A, p15A origin of replication. The primer sequences are shown again in Table S3 for better visibility.

Recently, we reported a RecET‐based markerless recombineering system for deletion and integration of large‐sized genes and clusters in *P. putida* (Choi *et al*., 2018). Although *P. putida* has chromosomes with high GC content (e.g. 61.6% in average for the chromosome of *P. putida* KT2440), conventional recombineering systems for *P. putida* chromosome modification use Bet recombinase derived from λ Red system (Chen *et al*., 2016; Cook *et al*., 2018; Sun *et al*., 2018), which is efficient in invading double‐stranded DNAs (dsDNAs) with low GC content (< 20%) (Rybalchenko *et al*., 2004), or a Bet recombinase homolog Ssr from *P. putida* DOT‐T1E (Aparicio *et al*., 2016). Thus, we exploited *Escherichia coli* Rac prophage‐derived RecET system (Choi *et al*., 2018) of which dsDNA invasion activity is independent of GC content of target loci (Noirot and Kolodner, 1998) considering the high GC content of *P. putida*. In addition, inducible expression of RecET proteins – of which leaky expression might reduce genome stability – from a RecET vector pJB658‐recET (Table S1) was tightly regulated by the P_m_/XylS system that requires *m*‐toluic acid for the induction (Choi *et al*., 2018). Once the expression of RecET proteins was induced, introduction of linear donor dsDNAs encoding antibiotic resistance markers [i.e. the *tetA(C)* and *aph(3ʹ)‐IIa* genes for tetracycline and kanamycin resistance, respectively] flanked by a pair of 100‐bp homologies to target chromosomal loci (Fig. 1A) was enough to integrate the antibiotic resistance markers to the target chromosomal loci. Moreover, a pair of mutant *loxP* sites (i.e. *lox71* and *lox66*) placed at both ends of the antibiotic resistance marker [e.g. *lox71‐tetA(C)‐lox66* cassette] allowed simple excision of the antibiotic resistance marker integrated to the chromosome upon the induction of Cre protein from the Cre vector pRK2Cre (Table S1) using isopropyl β‐d‐1‐thiogalactopyranoside (IPTG). A mutant *loxP* site (i.e. *lox72*) left behind the excision of the antibiotic resistance marker is extremely inefficient in the Cre‐induced recombination with *lox71* or *lox66* to be used for subsequent recombineering cycles (Lambert *et al*., 2007), allowing repeated use of the system for sequential modification of multiple target sites without significant interaction between engineered loci. Furthermore, instability of plasmid pJB658‐recET (Table S1) enabled its rapid curing upon simple streaking of recombinant strains harbouring the RecET vector (mediating ampicillin resistance) on LB‐agar medium without ampicillin (Choi *et al*., 2018). Similarly, temperature sensitivity of the plasmid pRK2Cre (Table S1) allowed its curing upon streaking recombinant strains harbouring the Cre vector (with kanamycin resistance) on LB‐agar medium without kanamycin followed by incubation at 37°C. As a result, recombineered strains could be easily converted to markerless and plasmid‐less strains (Choi *et al*., 2018), which is ideal for subsequent engineering and also prevents dissemination of antibiotic resistance genes to the environment (Jiang *et al*., 2017) upon accidental release of industrial strains. Using the RecET recombineering system, we demonstrated markerless deletion of chromosomal loci of *P. putida* KT2440 as large as 101.7 kb (i.e. 1.6% of *P. putida* KT2440 chromosome) in a single round of recombineering (Table S2) (Choi *et al*., 2018).

To establish a markerless recombineering system for the integration of heterologous BGCs to *P. putida* chromosome, we also devised a donor plasmid system (Choi *et al*., 2018). Most BGCs of heterologous natural products are longer than 6 kb (Loeschcke and Thies, 2015). However, the efficiency of gene integration via recombineering with linear donor dsDNA dramatically decreases as the insert size increases above 2–3.5 kb (Kuhlman and Cox, 2010), which includes the antibiotic resistance genes for selection [e.g. 1.7 kb *lox71‐tetA(C)‐lox66* cassette]. In addition, increased length of linear donor dsDNA reduces the amplification efficiency while elevating the mutation rate during PCR. Moreover, transformation efficiency decreases together as the size of DNA increases (Hanahan, 1983; Ohse *et al*., 1995; Sheng *et al*., 1995). Thus, a donor plasmid system that does not replicate in *P. putida* was developed to overcome such issues (Choi *et al*., 2018). Donor DNAs in forms of plasmids can be maintained stably with extremely low mutation rates in cloning hosts and easily isolated in large quantities from them. Supercoiling of the isolated donor plasmid also enhances transformation efficiency during recombineering. The recombineering efficiency can be further enhanced by increasing the length of homology arms (HAs) in the donor plasmid. Furthermore, the *sacB* gene was placed next to the origin of replication (e.g. pUC *ori* not replicating in *P. putida*) in the donor plasmid to combine two‐step double crossing‐over procedure into a single step (Choi *et al*., 2018). Briefly, integration of entire donor plasmid to the target chromosomal locus via a single cross‐over event on either HAs results in the expression of levansucrase from the *sacB* gene and kills the cell in the presence of sucrose and no NaCl. Thus, only recombinant cells that experienced double cross‐over on both HAs, harbour the genes/clusters to be integrated and the *lox71‐tetA(C)‐lox66* cassette, and lost the *sacB* gene and *ori* survive, while unmodified cells are selected out due to the supplementation of appropriate antibiotic [e.g. tetracycline if the donor plasmid harbours the *lox71‐tetA(C)‐lox66* cassette]. Knocking out 1‐kb region on the *pvdD* gene using 1 μg of a donor plasmid pTetSac‐ΔpvdD (Table S1) resulted in tens of times more recombinant strains with the *pvdD* gene knockout compared to when using 1 μg of linear donor dsDNA ΔpvdD1k100::tetA‐lox, demonstrating the capacity of the donor plasmid system in improving the recombineering efficiency (Choi *et al*., 2018). Cloning of 7.4 kb heterologous violacein BGC to an adaptor plasmid pTetSac‐ΔpvdD::Adaptor (Table S1) to construct a donor plasmid pTetSac‐ΔpvdD::Violacein (Table S1) and subsequent markerless recombineering of *P. putida* KT2440 harbouring the RecET vector pJB658‐recET (Table S1) using the donor plasmid resulted in markerless integration of the 7.4 kb heterologous BGC to the target locus (i.e. the *pvdD* gene) on *P. putida* KT2440 chromosome (Table S2) with successful biosynthesis of violacein (Choi *et al*., 2018).

In response to the request to provide more detailed protocols for our *P. putida* recombineering system, we report step‐by‐step procedures for the construction of linear donor dsDNAs and donor plasmids as well as recombineering *P. putida* with the RecET‐based markerless recombineering system using the donor DNAs constructed. In addition, examples of engineering *P. putida* with the recombineering protocol from our previous report are briefly summarized.

## Protocols

### Selection of recombineering targets for gene knockout and integration (1–3 h)

Chromosomal loci and episomal loci on single‐copy megaplasmids (e.g. genes on a TOL plasmid pWW0 of *P. putida* mt‐2 strain) to be deleted or used as platform sites for gene integration should be selected before proceeding to recombineering. In addition, DNA sequence of HAs and primers for HA construction should be prepared at this step. Two different procedures are used depending on the type of donor DNAs used for recombineering: linear donor dsDNAs for gene knockout (option A) and donor plasmids for gene knockout or integration (option B). For knocking out target genes, use of linear donor dsDNA accelerates recombineering procedure as its preparation steps are simpler while use of donor plasmid guarantees efficient and successful gene knockout.

#### Designing linear donor dsDNAs for gene knockout

- For each gene/region to be knocked out, define actual region to be deleted: either partial (e.g. in‐frame or out‐of‐frame deletion for truncation) or complete deletion of the target gene (Fig. 1A).
- Retrieve DNA sequences for a pair of 100‐bp regions neighbouring each deletion target. These sequences will be used to design HAs (Fig. 1A). It should be noted that HA of 50 bp is insufficient to engineer target sites on the *P. putida* chromosome.
- Design primer sets to amplify the *lox71‐tetA(C)‐lox66* cassette that is flanked by the 100‐bp HA pair (designed in step ii) from plasmid pTetAmulox (Tables S1, S3 and Fig. 1A). For example, two pairs of ˜70‐nt primers can be designed as follows: Each primer in the first pair (primers LinearDonor+ and LinearDonor−) consists of a ˜20‐nt segment complementarily binding to each end of the *lox71‐tetA(C)‐lox66* cassette and a ˜50‐nt segment from each inner half of the 100‐bp HA (Table S3 and Fig. 1A). Each primer in the second pair (primers LinearDonor++ and LinearDonor−−) consists of a ˜20‐nt segment from the 5ʹ ends of each primer in the first pair and a ˜50‐nt segment spanning the rest half of each 100‐bp HA (Table S3 and Fig. 1A). Melting temperature (*T*_m_) of each ˜20‐nt segment for primer binding is recommended to be above 60°C to facilitate amplification by PCR.
- Design primers for confirming gene knockouts. For example, a pair of primers binding outside the HA with their 3ʹ ends oriented towards the deletion target (primers Check+ and Check−) are good choices (Table S3). In addition, a pair of primers binding inside the *tetA(C)* gene with their 3ʹ ends oriented towards each near end of the gene [e.g. primers tetA(C)+ and tetA(C)−] are also useful (Table S3).
- Order the synthesis of the designed primers.

#### Designing donor plasmids for gene knockout and adaptor plasmids for gene integration

- Define actual regions to be deleted for gene knockout or integration.
- Retrieve a pair of ˜1‐kb DNA sequences flanking each deletion target, which will be used to design HA (Fig. 1B).
- Design two sets of primers (primer sets HA_L+/HA_L− and HA_R+/HA_R−) that will be used to amplify the pair of ˜1‐kb HA from the genomic DNA (gDNA) of *P. putida* (Table S3 and Fig. 1B). In addition, design two additional pairs of primers to amplify the (MCS−)*lox71‐tetA(C)‐lox66* (primer pair lox71+/lox66− or MCS+/lox66−) and the *sacB‐ori* (primers sacB+ and ori−) cassettes respectively (Table S3 and Fig. 1B). Use plasmid pTetSac‐ΔpvdD (Table S1) as a template to amplify the *lox71‐tetA(C)‐lox66* cassette and plasmid pTetSac‐ΔpvdD::Adaptor (Table S1) as a template to amplify the MCS‐*lox71‐tetA(C)‐lox66* cassette (Fig. 1B). The primers may have additional sequences at the 5ʹ ends that provide homologies for Gibson assembly or restriction recognition sites for cloning by restriction enzyme digestion and subsequent ligation (Table S3 and Fig. 1B). *T*_m_ of each primer binding sequence is recommended to be above ˜60°C to facilitate amplification by PCR as gDNA of *P. putida* has high GC content (e.g. 61.6% in average for *P. putida* KT2440).
- Design primers for confirming gene knockout and integration. For example, a pair of primers binding outside the HA with their 3ʹ ends oriented towards the deletion target are good choices (primers Check+ and Check−). In addition, a pair of primers binding inside the *tetA(C)* gene with their 3ʹ ends oriented towards each near end of the gene [e.g. primers tetA(C)+ and tetA(C)−] are also useful.
- Order the synthesis of the designed primers.

### Preparation of linear donor DNA for gene knockout (5–6 h)

- Amplify the *lox71‐tetA(C)‐lox66* cassette from plasmid pTetAmulox (Table S1) using primers LinearDonor+ and LinearDonor− (Table S3). The amplified product contains ˜50‐nt HA at each end (Fig. 1A).
- Purify the amplified product after resolving by gel electrophoresis followed by gel extraction.
- Amplify the purified product using primers LinearDonor++ and LinearDonor−− (Table S3), extending each end with the rest of the 100‐nt HA. This results in the *lox71‐tetA(C)‐lox66* cassette flanked by a pair of 100‐nt HAs (Fig. 1A). Amplification in large quantity is recommended as more than 10 μg of the product is desired.
- Purify the amplified product (i.e. linear donor dsDNA) and elute using deionized water. Elution at high concentration (> 300 ng μl^−1^) is preferred as > 3 μg of linear donor DNA is used for each recombineering procedure.

### Construction and preparation of donor plasmids for gene knockout and integration (2–3 and 4–6 days to prepare donor plasmids for gene knockout and integration, respectively)

- Amplify each components of the donor/adaptor plasmid by PCR (Fig. 1B). For example, amplify the HA pair from *P. putida* gDNA using primer sets HA_L+/HA_L− and HA_R+/HA_R− (Table S3 and Fig. 1B) and the MCS‐*lox71‐tetA(C)‐lox66* cassette from plasmid pTetSac‐ΔpvdD::Adaptor (Table S1) using primers MCS+ and lox66− (Table S3 and Fig. 1B). For gene knockout, however, amplification (and subsequent use) of the *lox71‐tetA(C)‐lox66* cassette, rather than the MCS‐*lox71‐tetA(C)‐lox66* cassette, from plasmid pTetAmulox (Table S1) using primers lox71+ and lox66− (Table S3) is recommended as repeated use of the MCS‐*lox71‐tetA(C)‐lox66* cassette unnecessarily introduces multiple MCS sites to the chromosome. In addition, the *sacB‐ori* cassette harbouring an origin of replication with low copy number [e.g. *sacB‐ori* (p15A) cassette from plasmid pTetSac15‐ΔpvdD2::Adaptor], rather than the *sacB‐ori* (pUC) cassette from plasmid pTetSac‐ΔpvdD::Adaptor (Table S1), may be amplified using primers sacB+ and ori− (Table S3 and Fig. 1B). This is a useful strategy when constructing an adaptor plasmid for cloning genes/BGCs of which cloning in high copy number plasmids and subsequent (leaky) expression burdens the cloning host and interrupts cloning/maintenance of the resulting donor plasmids.
- Assemble the four amplified products (i.e. two HAs, (MCS‐)*lox71‐tetA(C)‐lox66* cassette, and *sacB‐ori* cassette) to construct the donor plasmid for gene knockout or adaptor plasmid for the cloning genes of interest (Fig. 1B). For example, the two HAs and the (MCS‐)*lox71‐tetA(C)‐lox66* cassette can be assembled by overlapping PCR using primers HA_L+ and HA_R− (Table S3). The amplified product and the *sacB‐ori* cassette can be subsequently joined by Gibson assembly (Gibson *et al*., 2009). Alternatively, restriction enzyme digestion followed by ligation may be used if the primers used for the amplification contain appropriate restriction recognition sites. Of course, certain variations can be made to facilitate the cloning procedure depending on their own lab protocols. Transform a cloning host (e.g. *E. coli* DH5α) and screen cloned colonies on LB‐agar medium supplemented with 10 μg ml^−1^ tetracycline at 37°C.
- Identify a construct (i.e. donor plasmid for gene knockout or adaptor plasmid for gene integration) with correct sequence, especially sequences for the MCS, *lox71*, and *lox66*. Store cell stock with the correct construct for further use. To prepare donor plasmids for gene knockout, directly go to step vi.
- Inoculate cells harbouring a correctly constructed adaptor plasmid into 5 ml of LB medium supplemented with 10 μg ml^−1^ tetracycline, incubate at 37°C with rotary shaking at 200 rpm and isolate the adaptor plasmid to clone genes of interest to be integrated to *P. putida* chromosome.
- Amplify the gene(s)/cluster(s) of interest using primers possessing appropriate restriction recognition sites at their 5ʹ ends. Digest the amplified product(s) and the adaptor plasmid isolated in step iv using appropriate restriction enzymes. Ligate the digested DNAs together to construct donor plasmid for gene integration. Alternatively, the primers for target gene amplification may possess homologies to the cloning site for direct assembly of the amplified product(s) and the digested adaptor plasmid by Gibson assembly. Any other variations can be used to facilitate the cloning procedure. Transform a cloning host with the assembly product (e.g. *E. coli* DH5α), screen colonies harbouring correct construct (i.e. donor plasmid for gene integration) on LB‐agar medium supplemented with 10 μg ml^−1^ tetracycline at 37°C and identify colonies harbouring constructs with correct sequences. Store cell stocks for further use.
- To isolate donor plasmids for recombineering, inoculate cells harbouring the donor plasmids into 5–10 ml of LB medium supplemented with 10 μg ml^−1^ tetracycline, incubate at 37°C with rotary shaking at 200 rpm and isolate the donor plasmid using deionized water for the elution. Preparation of more than 3 μg of plasmids at concentrations higher than> 100 ng μl^−1^ is preferred.

### Recombineering of *P. putida* for gene knockout and integration (shortest 4N + 1 day for N cycles)

**Figure 2:**
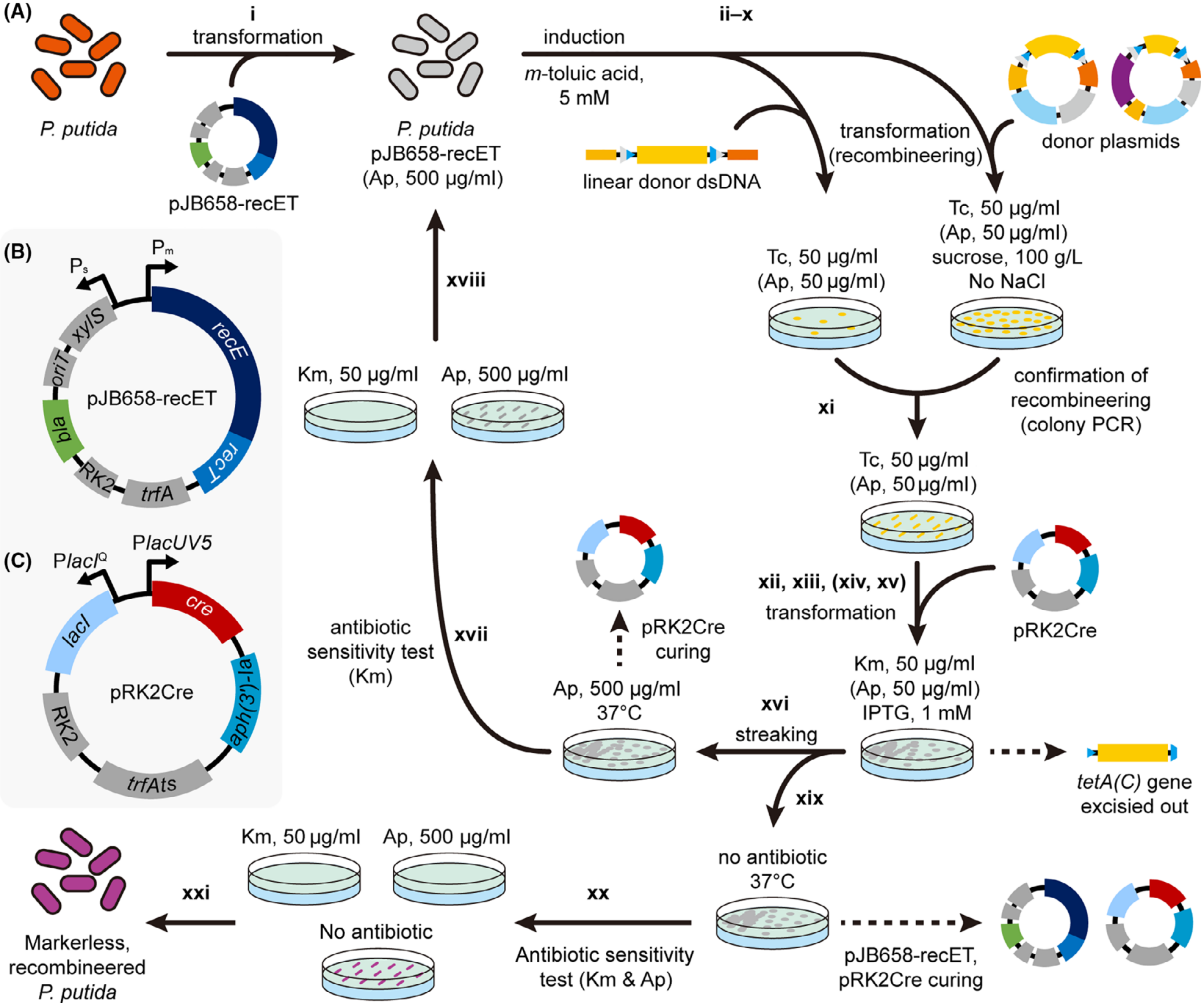
Scheme of markerless recombineering of *P. putida* using the RecET and Cre vectors. This figure shows more detailed procedures of what we previously described (Choi *et al*., 2018). A. Overall procedure of markerless recombineering of *P. putida* described in subsection ‘*Recombineering of* P. putida *for gene knockout and integration*’ of protocols. Bold numbers indicate corresponding steps of the protocol. Refer to the steps in the protocol for detailed description. For steps x to xv, ampicillin may be added to the plates to maintain the RecET vector for subsequent rounds of recombineering. Dashed lines indicate removal of corresponding element from recombinant strains during incubation. Ap, ampicillin; Km, kanamycin; Tc, tetracycline. B. Vector map of the RecET vector pJB658‐recET. *bla*, ampicillin resistance gene; RK2, RK2 origin of replication; *trfA*, gene coding replicase of RK2 origin. C. Vector map of the Cre vector pRK2Cre. *aph(3ʹ)‐Ia*, kanamycin resistance gene; *trfAts*, temperature‐sensitive version of the *trfA* gene.
- Inoculate a single colony into 5 ml LB (5 g l^−1^ NaCl) medium supplemented with 500 μg ml^−1^ of ampicillin in 50 ml conical tube and incubate at 30°C for several hours with rotary shaking at 200 rpm. The screw cap of the conical tube is recommended to be slightly open and held with a small patch of adhesive tape for aeration. Meanwhile, prepare 50 ml of LB (5 g l^−1^ NaCl) medium supplemented with 500 μg ml^−1^ of ampicillin and 5 mM *m*‐toluic acid in 250 ml Erlenmeyer flask pre‐warmed at 30°C with rotary shaking at 200 rpm. *m‐*Toluic acid stock solution dissolved in ethanol at 1 M concentration may be used for the supplementation. Dissolution of *m*‐toluic acid to the medium can be facilitated by static incubation of the medium supplemented with *m*‐toluic acid at 42°C with brief and intermittent manual shaking. Pre‐warming the medium above 30°C can also facilitate the dissolution of *m*‐toluic acid upon the supplementation.
- Transfer 1 ml of the culture with full growth – optical density measured at 600 nm (OD_600_) should reach ˜5 – to the pre‐warmed medium and incubate at 30°C with rotary shaking at 200 rpm until OD_600_ reaches ˜2 to induce RecET protein expression (Fig. 2A). This step usually takes ˜5 h, but the incubation time may depend on *P. putida* strains and physiological status of the overnight culture.
- Ice‐chill the culture for 5 min (on ice with gaps filled with water) and harvest cells from 49 ml of the chilled culture by centrifugation at 2090 *g* for 10 min.
- Wash the pellet from each 49 ml culture twice by resuspension to 2 ml of ice‐cold 300 mM sucrose solution and subsequent centrifugation in two 1.5 ml microcentrifuge tubes at 4000 *g* for 2 min at 4°C. Centrifuge once more after two rounds of washing to completely remove the supernatant.
- Resuspend the washed pellet to 200 μl of ice‐cold 300 mM sucrose solution for each OD_600_ unit of the cultured cells (before the harvest). For example, resuspend the washed cell pellet from 49 ml of the culture with OD_600_ = 2.00 to 400 μl (=2.00 × 200 μl) of ice‐cold 300 mM sucrose solution.
- Aliquot 70 μl of the resuspended cells to 1.5‐ml microcentrifuge tubes kept on ice.
- Add 3 μg or more linear donor dsDNA or 1 μg or more donor plasmid to each fresh aliquot (Fig. 2A), mix by tapping and incubate on ice for 5 min.
- Electroporate the competent cell donor DNA mixture in 1‐mm cuvette by applying electric pulse of 1.8 kV with capacitance and resistance of 25 μF and 200 Ω, respectively. Immediately resuspend the transformants to 900 μl of LB (5 g l^−1^ NaCl) and recover at 30°C for 2 h without shaking.
- For gene knockout using linear donor dsDNAs, plate the recovered cells on LB‐agar plates supplemented with 50 μg ml^−1^ tetracycline (Fig. 2A). For gene knockout/integration using donor plasmids, plate the recovered cells on LB‐agar medium with 0 g l^−1^ NaCl, 100 g l^−1^ sucrose, and 50 μg ml^−1^ tetracycline (Fig. 2A). To maintain the RecET vector for subsequent iterative recombineering, 500 μg ml^−1^ ampicillin may be supplemented together. Incubate statically at 30°C until colonies grow on the plate. Recombineered colonies usually grow within overnight but may grow slower depending on the engineering targets and engineered status of the cells.
- Examine the gene knockout/integration by colony PCR using primer sets appropriate for the confirmation [e.g. primers Check+ and Check− or combinations with primers tetA(C)+ and tetA(C)−; Table S3] while making a master plate of the colonies for subsequent use (Fig. 2A). The sequence of the integrated genes may be optionally examined through amplification of the genes by colony PCR followed by sequencing of the amplified products.
- Inoculate a colony with desired modification into 2–5 ml of LB (5 g l^−1^ NaCl) medium supplemented with 50 μg ml^−1^ tetracycline and incubate at 30°C for several hours with rotary shaking at 200 rpm. Ampicillin (500 μg ml^−1^) may be added for stable maintenance of the RecET vector for additional rounds of recombineering.
- Harvest the cells, prepare competent cells and transform with plasmid pRK2Cre (Table S1 and Fig. 2C) following the description in step i (Fig. 2A). Plate the transformants on LB‐agar plate supplemented with 50 μg ml^−1^ of kanamycin and 1 mM IPTG to induce the expression of Cre protein and excise out the *tetA(C)* gene via site‐specific recombination between the *lox71* and *lox66* sites (Fig. 2A). Thus, tetracycline should not be added to the LB‐agar medium. Ampicillin (500 μg ml^−1^) may be supplemented together to maintain the RecET vector. Incubate the plates at 30°C until kanamycin‐resistant colonies grow. This usually takes around one full day.
- (Optional) To check the excision of the *tetA(C)* gene based on tetracycline resistance/sensitivity, streak the kanamycin‐resistant colonies serially on LB‐agar medium supplemented with 50 μg ml^−1^ tetracycline and LB‐agar medium supplemented with 50 μg ml^−1^ kanamycin. Ampicillin (500 μg ml^−1^) may be added together to maintain plasmid pJB658‐recET. Incubate the plates at 30°C until cell growth is observed from the kanamycin plate. This step usually takes several hours. This step may be skipped as the efficiency of the *tetA(C)* gene excision upon Cre protein induction reaches 100% (Choi *et al*., 2018).
- Cells growing on the kanamycin plate while not growing on the tetracycline plate are ones without the *tetA(C)* gene upon the introduction of the Cre vector pRK2Cre. Colony PCR may be optionally conducted to further confirm the *tetA(C)* gene excision. To repeat additional recombineering cycles, move on to the next step. To finish *P. putida* recombineering, go to step xix.
- Streak colonies that lost the *tetA(C)* gene on LB‐agar medium supplemented with 500 μg ml^−1^ ampicillin and incubate at 37°C to isolate colonies cured of the Cre vector pRK2Cre (Fig. 2A). Several hours of incubation are enough to obtain single colonies.
- Check plasmid pRK2Cre curing by serially streaking the colonies on two LB‐agar media each supplemented with 50 μg ml^−1^ kanamycin and 500 μg ml^−1^ ampicillin, respectively (Fig. 2A). Incubate the plates at 30°C until cell growth is observed from the ampicillin plate. This usually takes several hours.
- Cells growing on the ampicillin plate while not growing on the kanamycin plate are ones cured of plasmid pRK2Cre. Go to step ii for additional rounds of recombineering (Fig. 2A).
- Streak colonies from step xv on LB‐agar medium without antibiotic and incubate at 37°C to isolate colonies cured of both the RecET vector pJB658‐recET and the Cre vector pRK2Cre (Fig. 2A). Several hours of incubation is enough to obtain single colonies.
- Check the curing of plasmid pRK2Cre by serially streaking the colonies on three LB‐agar media each supplemented with 500 μg ml^−1^ ampicillin, 50 μg ml^−1^ kanamycin, and no antibiotic (Fig. 2A). Incubate the plates at 30°C until cell growth is observed from the plate without antibiotic. This usually takes several hours.
- Cells not growing on the kanamycin plate are ones cured of the Cre vector. Store a cell stock of the recombineered strain and finish *P. putida* recombineering for gene knockout/integration (Fig. 2A).

- Transform *P. putida* strain that will be applied for gene knockout or integration with the RecET vector pJB658‐recET (Table S1 and Fig. 2A). Briefly, incubate *P. putida* strain in appropriate medium, such as 2–5 ml of LB medium with NaCl concentration reduced to 5 g l^−1^ [LB (5 g l^−1^ NaCl) medium], and conditions (e.g. 30°C, 200 rpm, with antibiotics, if required) to full density. This usually takes several hours. Harvest *P. putida* cells from 1 ml of the culture and wash the cells twice by resuspension to 1 ml of 300 mM sucrose solution and subsequent centrifugation at 4000 *g* for 2 min. Resuspend the pellet to 100 μl of 300 mM sucrose solution and add 0.1–1 μg of plasmid pJB658‐recET (Table S1 and Fig. 2B). Electroporate the cells in 1‐mm cuvette with electric pulse of 1.8 kV with capacitance and resistance of 25 μF and 200 Ω, respectievly. Recover the transformants in LB medium at 30°C and plate/streak the cells on LB‐agar medium supplemented with 500 μg ml^−1^ of ampicillin. Alternatively, *P. putida* strains already harbouring plasmid pJB658‐recET may be streaked on LB‐agar medium supplemented with 500 μg ml^−1^ of ampicillin to isolate fresh colonies. Incubate the plates at 30°C.

## Examples

**Figure 3:**
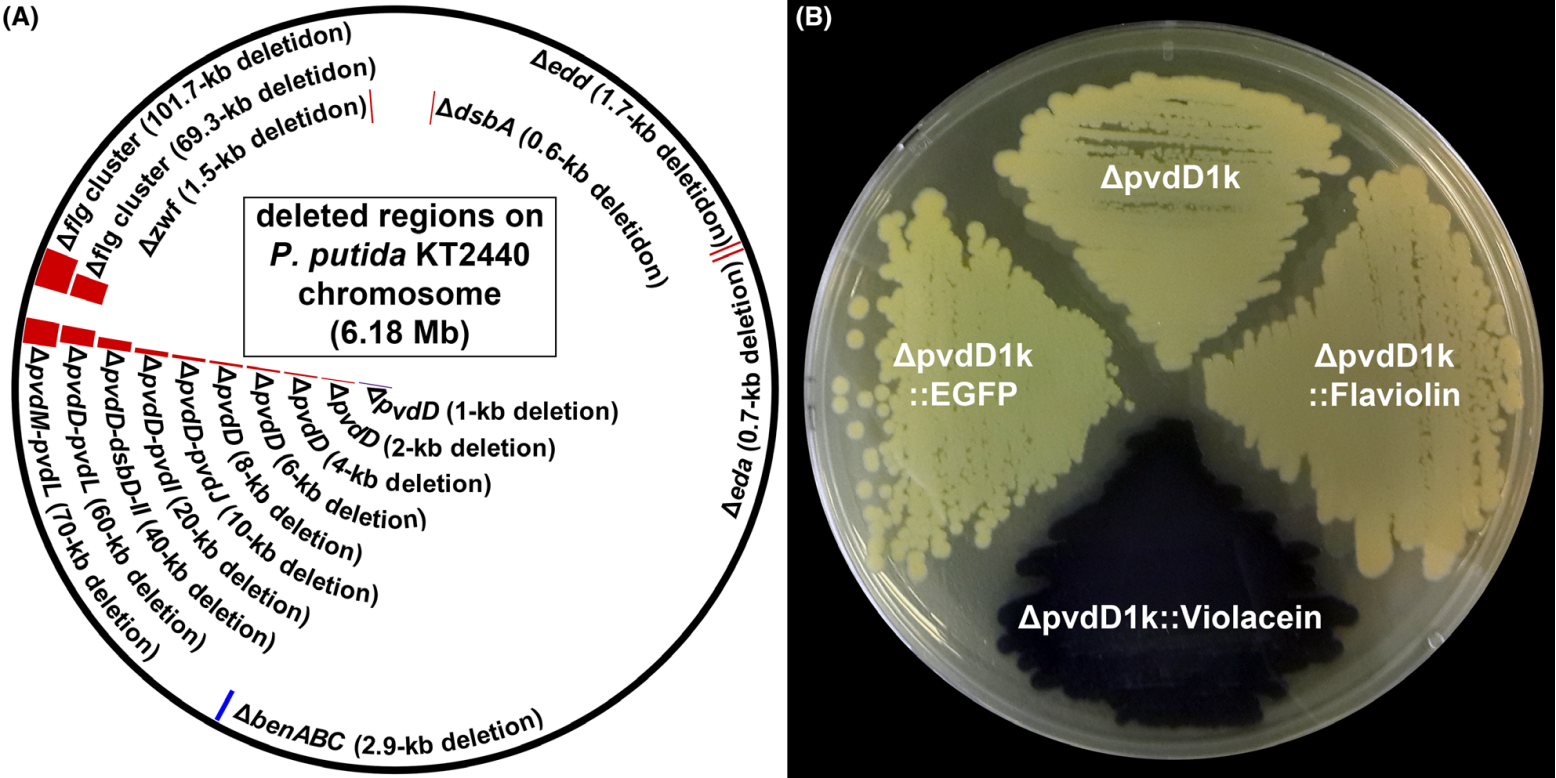
Examples of markerless recombineering in *P. putida*. A. Chromosomal regions of *P. putida* KT2440 examined for markerless deletion using the RecET recombineering system. Red, blue, and purple marks indicate regions knocked out using linear donor dsDNAs, donor plasmid, and both of them respectively. This figure includes the results of the previously reported (Choi *et al*., 2018) and updated genome engineering. B. Recombinant *P. putida* strains ΔpvdD::EGFP, ΔpvdD::Flaviolin, and ΔpvdD::Violacein harbouring heterologous biosynthetic genes/clusters of EGFP, flaviolin, and violacein on the chromosome (Table S2) show green, brown, and purple colours of each product respectively. These results have already been reported in our previous paper (Choi *et al*., 2018), but we took a new picture of engineered strains for better understanding of the protocols in this paper.

**Figure 4:**
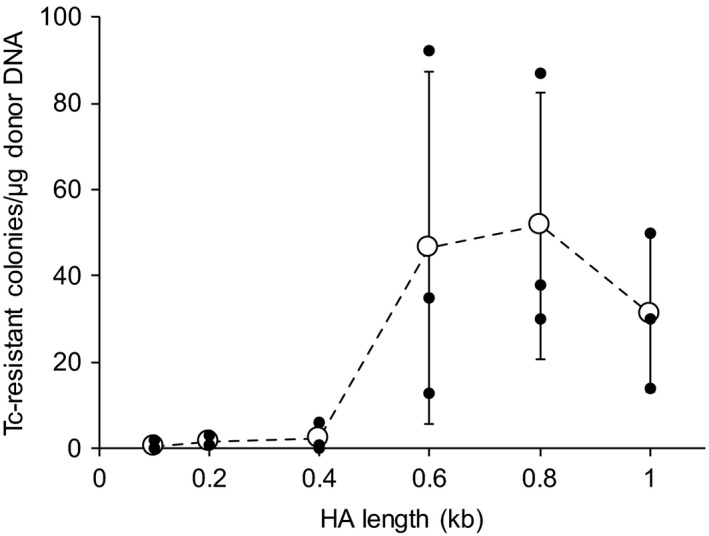
Effect of HA length on recombineering efficiency. The number of tetracycline (Tc)‐resistant colonies appeared after recombineering using 1 μg of donor plasmids pTetSac‐ΔpvdD100::Adaptor, pTetSac‐ΔpvdD200::Adaptor, pTetSac‐ΔpvdD400::Adaptor, pTetSac‐ΔpvdD600::Adaptor, pTetSac‐ΔpvdD800::Adaptor, and pTetSac‐ΔpvdD::Adaptor harbouring pairs of 0.1‐, 0.2‐, 0.4‐, 0.6‐, 0.8‐, and 1.0‐kb HAs respectively (Table S1). Successful knockout of the *pvdD* gene of all eight colonies randomly selected from each knockout experiment – or all colonies for experiments with less than eight colonies – was verified. The values and error bars represent means and standard deviations of colony counts from triplicate experiments, respectively, while all the actual three data points are also shown.

Markerless recombineering of *P. putida* KT2440 using linear donor dsDNAs with 100‐bp HA pairs successfully knocked out 16 different genes/regions throughout the chromosome (Choi *et al*., 2018). Briefly, 10 different regions (1.0, 2.0, 4.0, 6.0, 8.0, 10.0, 20.0, 40.0, 60.0 and 70.0 kb in length) centred at the *pvdD* gene, two different regions around the flagellar gene cluster (69.4 and 101.7 kb) and four other genes, including the *dsbA* (0.6 kb), *eda* (0.7 kb), *edd* (1.7 kb) and *zwf* (1.5 kb) genes, were deleted (Table S2 and Fig. 3A) (Choi *et al*., 2018). It should be noted that the 60.0‐, 69.4‐, 70.0‐, and 101.7‐kb regions deleted by single rounds of recombineering correspond to 0.97%, 1.1%, 1.1% and 1.6% of *P. putida* KT2440 chromosome (6181.9 kb). In addition, the *pvdD* gene could be more efficiently knocked out when a donor plasmid pTetSac‐ΔpvdD (Table S1) was used (Fig. 3A) instead of the linear donor dsDNA ΔpvdD1k100::tetA‐lox for recombineering (Choi *et al*., 2018). The efficient knockout of genes using donor plasmids was further demonstrated by successfully knocking out the *benABC* genes (2.9 kb) using another donor plasmid pTetSac‐ΔbenABC (Tables S1, S2 and Fig. 3A). To examine the effect of the length of HAs in donor plasmids on recombineering efficiency, the *pvdD* gene knockout experiments were conducted using a donor plasmid pTetSac‐ΔpvdD::Adaptor with a pair of 1‐kb HAs (Table S1) and a series of pTetSac‐ΔpvdD::Adaptor variants with pairs of 0.1‐, 0.2‐, 0.4‐, 0.6‐, and 0.8‐kb HAs (Table S1). The number of tetracycline‐resistant colonies appeared after each recombineering experiment drastically deceased as the length of HAs decreased from 0.6 kb to 0.4 kb (Fig. 4), while successful knockout of the *pvdD* gene was verified in all eight colonies randomly selected from each recombineering experiment – or entire colonies if less than eight colonies grew. These results indicate that the use of donor plasmids with HA pairs longer than 0.6 kb is crucial to guarantee efficient modification of target genes on the *P. putida* chromosome. Furthermore, heterologous biosynthetic genes/clusters of a fluorescent protein EGFP (1.2 kb), a polyketide flaviolin (1.2 kb), a terpenoid lycopene (3.5 kb), and an amino acid‐derived secondary metabolite violacein (7.4 kb) were efficiently integrated to the *pvdD* gene locus of *P. putida* KT2440 as directed by the HAs of corresponding donor plasmids pTetSac‐ΔpvdD::EGFP, pTetSac‐ΔpvdD::Flaviolin, pTetSac‐ΔpvdD::Lycopene, and pTetSac‐ΔpvdD::Violacein (Tables S1 and S2) (Choi *et al*., 2018). Among four resulting recombinant strains, ΔpvdD::EGFP, ΔpvdD::Flaviolin and ΔpvdD::Violacein strains (Table S2) showed green, brown, and purple colours of EGFP, flaviolin, and violacein, respectively (Fig. 3B), supporting successful biosynthesis of each product.

## Discussion

*Pseudomonas putida* has been considered as a promising host strain for the production of diverse secondary metabolites of importance (Loeschcke and Thies, 2015). However, engineering tools for the expression of such heterologous natural product BGCs in *P. putida* have not been optimal. In particular, most gene knockout tools have not been optimal for deleting large regions on the chromosome, and integration of heterologous BGCs has relied on either time‐consuming homologous recombination (7–10 days/cycle) (Cao *et al*., 2012; Martinez‐Garcia and de Lorenzo, 2012) or fast yet unpredictable transposon‐mediated random insertion (3–4 days/cycle) (Martinez‐Garcia and de Lorenzo, 2012; Loeschcke *et al*., 2013; Domrose *et al*., 2017). Thus, development of rapid and reliable tools for knocking out and integrating large‐sized genes/clusters had been demanded. Our markerless recombineering system for *P. putida*, of which detailed protocols are provided in this paper, allows rapid (4N + 1 days for N cycles of recombineering) and efficient deletion of chromosomal regions and integration of heterologous BGCs. Examples of recombineering include, but the capacity of the system is not limited to, knocking out 17 different regions of 0.6–101.7 kb and integrating heterologous BGCs of 1.2–7.4 kb (Choi *et al*., 2018), successfully demonstrating the use of the recombineering system in constructing plasmid/marker‐free overproducers of heterologous natural products. Engineering of *P. putida* using this recombineering system is expected to be expedited by adopting recent strategies of combining the recombinase and Cre vectors (i.e. plasmids pJB658‐recET and pRK2Cre in this system, respectively) (Song and Lee, 2013) and engineering of multiple loci simultaneously (Jensen *et al*., 2015; Cho *et al*., 2017), facilitating the study of *P. putida* and related species and streamlining the construction of high performance *P. putida* overproducer strains.

## Conflict of interest

None declared.

## Supporting information


**Table S1.** Plasmids used for markerless recombineering of *P. putida*.
**Table S2.** Examples of recombinant *P. putida* KT2440 strains generated by markerless recombineering.
**Table S3.** Examples of primers for markerless recombineering.
