# Supplementary material for: Protocols for RecET‐based markerless gene knockout and integration to express heterologous biosynthetic gene clusters in Pseudomonas putida
Source: Microb Biotechnol. 2019 Feb 14;13(1):199–209. doi: 10.1111/1751-7915.13374 (PMC6922525; doi:10.1111/1751-7915.13374)
Supplement: Supplementary file 1 — Table S1. Plasmids used for markerless recombineering of P. putida. Table S2. Examples of recombinant P. putida KT2440 strains generated by markerless recombineering. Table S3. Examples of primers for markerless recombineering. [file MBT2-13-199-s001.docx]

**Supporting Information for**

**Protocols for RecET-based markerless gene knockout and integration to express heterologous biosynthetic gene clusters in *Pseudomonas putida***

Kyeong Rok Choi^1^ and Sang Yup Lee^1,2,3,4^*

^1^Metabolic and Biomolecular Engineering National Research Laboratory, Systems Metabolic Engineering and Systems Healthcare Cross Generation Collaborative Laboratory, Department of Chemical and Biomolecular Engineering (BK21 Plus Program), Institute for the BioCentury, Korea Advanced Institute of Science and Technology (KAIST), 291 Daehak-ro, Yuseong-gu, Daejeon 34141, Republic of Korea

^2^BioProcess Engineering Research Center, KAIST, 291 Daehak-ro, Yuseong-gu, Daejeon 34141, Republic of Korea

^3^BioInformatics Research Center, KAIST, 291 Daehak-ro, Yuseong-gu, Daejeon 34141, Republic of Korea

^4^Novo Nordisk Foundation Center for Biosustainability, Technical University of Denmark, 2800 Kongens Lyngby, Denmark

*For correspondence. E-mail leesy@kaist.ac.kr; Tel. +82 42 350 3930; Fax +82 42 350 8800

Running title: *Pseudomonas putida* genome engineering

**Table S1. Plasmids used for markerless recombineering of *P. putida*.**

| **Plasmid** | **Description^a^** | **References** |
| --- | --- | --- |
| pJB658-recET | RecET vector;  pJB658 derivative, *E. coli* MG1655 *recET*; Ap^R^ | Choi *et al*. (2018) |
| pRK2Cre | Cre vector;  RK2*ts* origin (temperature-sensitive), *lacI^Q^*, P*lacUV5*, *cre*; Km^R^ | Choi *et al*. (2018) |
| pTetAmulox | Template vector for linear donor dsDNAs;  *lox66-tetA(C)-lox77*; Ap^R^, Tc^R^ | Choi *et al*. (2018) |
| pTetSac-ΔpvdD | Donor plasmid for knocking out the *pvdD* gene of *P. putida* KT2440;  pUC origin, *sacB*, *lox71-tetA(C)*-*lox66*, a pair of 1-kb homology arms for the *pvdD* gene of *P. putida* KT2440; Tc^R^ | Choi *et al*. (2018) |
| pTetSac-ΔbenABC | Donor plasmid for knocking out the *benABC* genes of *P. putida* KT2440;  pUC origin, *sacB*, *lox71-tetA(C)*-*lox66*, a pair of 1-kb homology arms for the *benABC* genes of *P. putida* KT2440; Tc^R^ | This study |
| pTetSac-ΔpvdD::Adaptor | Adaptor plasmid for cloning genes to be integrated to the *pvdD* gene locus of *P. putida* KT2440;  pUC origin, *sacB*, *lox71-tetA(C)*-*lox66*, a pair of 1-kb homology arms for the *pvdD* gene of *P. putida* KT2440, a multiple cloning site; Tc^R^ | Choi *et al*. (2018) |
| pTetSac-ΔpvdD100::Adaptor | Adaptor plasmid for cloning genes to be integrated to the *pvdD* gene locus of *P. putida* KT2440;  pUC origin, *sacB*, *lox71-tetA(C)*-*lox66*, a pair of 0.1-kb homology arms for the *pvdD* gene of *P. putida* KT2440, a multiple cloning site; Tc^R^ | This study |
| pTetSac-ΔpvdD200::Adaptor | Adaptor plasmid for cloning genes to be integrated to the *pvdD* gene locus of *P. putida* KT2440;  pUC origin, *sacB*, *lox71-tetA(C)*-*lox66*, a pair of 0.2-kb homology arms for the *pvdD* gene of *P. putida* KT2440, a multiple cloning site; Tc^R^ | This study |
| pTetSac-ΔpvdD400::Adaptor | Adaptor plasmid for cloning genes to be integrated to the *pvdD* gene locus of *P. putida* KT2440;  pUC origin, *sacB*, *lox71-tetA(C)*-*lox66*, a pair of 0.4-kb homology arms for the *pvdD* gene of *P. putida* KT2440, a multiple cloning site; Tc^R^ | This study |
| pTetSac-ΔpvdD600::Adaptor | Adaptor plasmid for cloning genes to be integrated to the *pvdD* gene locus of *P. putida* KT2440;  pUC origin, *sacB*, *lox71-tetA(C)*-*lox66*, a pair of 0.6-kb homology arms for the *pvdD* gene of *P. putida* KT2440, a multiple cloning site; Tc^R^ | This study |
| pTetSac-ΔpvdD800::Adaptor | Adaptor plasmid for cloning genes to be integrated to the *pvdD* gene locus of *P. putida* KT2440;  pUC origin, *sacB*, *lox71-tetA(C)*-*lox66*, a pair of 0.8-kb homology arms for the *pvdD* gene of *P. putida* KT2440, a multiple cloning site; Tc^R^ | This study |
| pTetSac15-ΔpvdD2::Adaptor | Adaptor plasmid for cloning genes to be integrated to the *pvdD* gene locus of *P. putida* KT2440;  p15A origin, *sacB*, *lox71-tetA(C)*-*lox66*, a pair of 1-kb homology arms for the *pvdD* gene of *P. putida* KT2440, a multiple cloning site; Tc^R^ | This study |
| pTetSac-ΔpvdD::EGFP | Donor plasmid for integrating the *egfp* gene to the *pvdD* gene locus of *P. putida* KT2440;  pTetSac-ΔpvdD::Adaptor derivative, P*tac*-*egfp-rrnB* T1T2 terminator; Tc^R^ | Choi *et al*. (2018) |
| pTetSac-ΔpvdD::Flaviolin | Donor plasmid for integrating flaviolin biosynthetic gene *rppA* to the *pvdD* gene locus of *P. putida* KT2440;  pTetSac-ΔpvdD::Adaptor derivative, P*tac*-*rppA* (*S. griseus*); Tc^R^ | Choi *et al*. (2018) |
| pTetSac-ΔpvdD::Lycopene | Donor plasmid for integrating lycopene biosynthetic gene cluster *crtEIB* to the *pvdD* gene locus of *P. putida* KT2440;  pTetSac-ΔpvdD::Adaptor derivative, P*gntT104*-*crtEIB*; Tc^R^ | Choi *et al*. (2018) |
| pTetSac-ΔpvdD::Violacein | Donor plasmid for integrating violacein biosynthetic gene cluster *vioABCDE* to the *pvdD* gene locus of *P. putida* KT2440;  pTetSac-ΔpvdD::Adaptor derivative, P*tac-vioABCDE*, no ribosome binding site between P*tac* and *vioA*; Tc^R^ | Choi *et al*. (2018) |

^a^Ap, ampicillin; Km, kanamycin; Tc, tetracycline, ^R^, resistant.

**Table S2. Examples of recombinant *P. putida* KT2440 strains generated by markerless recombineering.**

| **Strains** | **Description^a^** | **References** |
| --- | --- | --- |
| ΔpvdD1k | KT2440 Δ*pvdD* (1-kb deletion) | Choi *et al*. (2018) |
| ΔpvdD2k | KT2440 Δ*pvdD* (2-kb deletion) | Choi *et al*. (2018) |
| ΔpvdD4k | KT2440 Δ*pvdD* (4-kb deletion) | Choi *et al*. (2018) |
| ΔpvdD6k | KT2440 Δ*pvdD* (6-kb deletion) | Choi *et al*. (2018) |
| ΔpvdD8k | KT2440 Δ*pvdD* (8-kb deletion) | Choi *et al*. (2018) |
| ΔpvdD10k | KT2440 Δ*pvdD-pvdJ* (10-kb deletion) | Choi *et al*. (2018) |
| ΔpvdD20k | KT2440 Δ*pvdD-pvdI* (20-kb deletion) | Choi *et al*. (2018) |
| ΔpvdD40k | KT2440 Δ*pvdD-dsbD-II* (40-kb deletion) | Choi *et al*. (2018) |
| ΔpvdD60k | KT2440 Δ*pvdD-pvdL* (60-kb deletion) | Choi *et al*. (2018) |
| ΔpvdD70k | KT2440 Δ*pvdM-pvdL* (70-kb deletion) | Choi *et al*. (2018) |
| Δflg69.3k | KT2440 Δ*PP_4329-ycgR* (69.3-kb deletion) | Choi *et al*. (2018) |
| Δflg101.7k | KT2440 Δ*PP_4329-PP_4424* (69.3-kb deletion) | Choi *et al*. (2018) |
| Δeda | KT2440 Δ*eda* | Choi *et al*. (2018) |
| Δedd | KT2440 Δ*edd* | Choi *et al*. (2018) |
| ΔdsbA | KT2440 Δ*dsbA* | Choi *et al*. (2018) |
| Δzwf | KT2440 Δ*zwf* | Choi *et al*. (2018) |
| ΔbenABC | KT2440 Δ*benABC* | This study |
| ΔpvdD::EGFP | KT2440 Δ*pvdD* (1-kb deletion)*::*P*tac-egfp-rrnB T1T2* terminator | Choi *et al*. (2018) |
| ΔpvdD::Flaviolin | KT2440 Δ*pvdD* (1-kb deletion)*::*P*tac-rppA* | Choi *et al*. (2018) |
| ΔpvdD::Lycopene | KT2440 Δ*pvdD* (1-kb deletion)*::*P*gntT104-crtEIB* | Choi *et al*. (2018) |
| ΔpvdD::Violacein | KT2440 Δ*pvdD* (1-kb deletion)*::*P*tac-vioABCDE*, no ribosome binding site between P*tac* and *vioA* | Choi *et al*. (2018) |

^a^Ap, ampicillin; Km, kanamycin; Tc, tetracycline, ^R^, resistant.

**Table S3. Examples of primers for markerless recombineering.**

| **Primers** | **Sequences^a^** | **Templates** |
| --- | --- | --- |
| LinearDonor+ | N_~50_CAGCTGAAGCTTTACCGTTC | Plasmid pTetAmulox |
| LinearDonor- | N_~50_GATCTGATGGGTACCGTTCG |  |
| LinearDonor++ | N_~50_N_~20_ | PCR products amplified with primers LinearDonor+/- |
| LinearDonor-- | N_~50_N_~20_ |  |
| Check+ | N_~20_ | Recombineered *P. putida* gDNA (from colonies) |
| Check- | N_~20_ |  |
| tetA(C)+ | CGCTCTGGGTCATTTTCGG |  |
| tetA(C)- | CGCTCATGAGCCCGAAGTG |  |
| HA_L+ | N_n_N_~20_ | *P. putida* gDNA |
| HA_L- | N_n_N_~20_ |  |
| HA_R+ | N_n_N_~20_ | *P. putida* gDNA |
| HA_R- | N_n_N_~20_ |  |
| lox71+ | N_n_CAGCTGAAGCTTTACCGTTC | Plasmid pTetSac-ΔpvdD |
| lox66- | N_n_GATCTGATGGGTACCGTTCG |  |
| MCS+ | N_n_CGGGCCCTTAATTAAGGCC | Plasmid pTetSac-ΔpvdD::Adaptor |
| lox66- | N_n_GATCTGATGGGTACCGTTCG |  |
| sacB+ | N_n_ATCCTTTTTAACCCATCAC | Plasmid pTetSac-ΔpvdD::Adaptor or pTetSac15-ΔpvdD2::Adaptor |
| ori- | N_n_TATAAACGCAGAAAGGCCCAC |  |

^a^N_~50_, appropriate sequence corresponding to ~50-nt half of homology arms designed as described in the protocols and Fig. 1A; N_~20_, appropriate sequences of ~20 nt with T_m_ above ~60°C designed as described in the protocol and Fig. 1; N_n_, appropriate sequences coding homologies for Gibson assembly or restriction recognition sites designed as described in the protocols and Fig. 1B.

**Reference**

Choi, K.R., Cho, J.S., Cho, I.J., Park, D., and Lee, S.Y. (2018) Markerless gene knockout and integration to express heterologous biosynthetic gene clusters in *Pseudomonas putida*. *Metab Eng* **47**: 463-474.
